# Plastid Genome of *Equisetum xylochaetum* from the Atacama Desert, Chile and the Relationships of *Equisetum* Based on Frequently Used Plastid Genes and Network Analysis

**DOI:** 10.3390/plants11071001

**Published:** 2022-04-06

**Authors:** Anchittha Satjarak, Linda E. Graham, Marie T. Trest, Patricia Arancibia-Avila

**Affiliations:** 1 Plants of Thailand Research Unit, Department of Botany, Faculty of Science, Chulalongkorn University, Bangkok 10330, Thailand; 2 Department of Botany, University of Wisconsin, Madison, WI 53706-1313, USA; lkgraham@wisc.edu (L.E.G.); marie.trest@wisc.edu (M.T.T.); 3 Department of Basic Sciences, University of Bío-Bío, Chillan 3780000, Chile; parancib@ubiobio.cl

**Keywords:** *Equisetum*, plastid genome, haplotype map

## Abstract

The modern pteridophyte genus *Equisetum* is the only survivor of Sphenopsida, an ancient clade known from the Devonian. This genus, of nearly worldwide distribution, comprises approximately 15 extant species. However, genomic information is limited. In this study, we assembled the complete chloroplast genome of the giant species *Equisetum xylochaetum* from a metagenomic sequence and compared the plastid genome structure and protein-coding regions with information available for two other *Equisetum* species using network analysis. *Equisetum* chloroplast genomes showed conserved traits of quadripartite structure, gene content, and gene order. Phylogenetic analysis based on plastome protein-coding regions corroborated previous reports that *Equisetum* is monophyletic, and that *E. xylochaetum* is more closely related to *E. hyemale* than to *E. arvense*. Single-gene phylogenetic estimation and haplotype analysis showed that *E. xylochaetum* belonged to the subgenus *Hippochaete*. Single-gene haplotype analysis revealed that *E. arvense*, *E. hyemale*, *E. myriochaetum*, and *E. variegatum* resolved more than one haplotype per species, suggesting the presence of a high diversity or a high mutation rate of the corresponding nucleotide sequence. Sequences from *E. bogotense* appeared as a distinct group of haplotypes representing the subgenus *Paramochaete* that diverged from *Hippochaete* and *Equisetum*. In addition, the taxa that were frequently located at the joint region of the map were *E. scirpoides* and *E. pratense*, suggesting the presence of some plastome characters among the *Equiseum* subgenera.

## 1. Introduction

*Equisetum* L. is a genus of vascular plants that represents ancient Sphenopsida, a long-enduring clade known from fossils of the Devonian and later ages and, therefore, is considered useful in understanding the evolution of vascular plants. This genus is comprised of approximately 15 extant species, with a nearly worldwide distribution [1,2]. Previous studies have examined the evolutionary relationships among stem and crown *Equisetum* species using both morphology and genomic data. However, because morphology can vary as the result of hybridization and climate differences, molecular approaches have become popular. Recent studies have indicated three subgenera, including the primitive subgenus *Paramochaete*, and the later diverging subgenera *Equisetum* and *Hippochaete*. However, such relationships were estimated from relatively few plastid genes, e.g., *rbcL*, *rps4*, and *trnL-F* e.g., [3,4,5,6].

Among reported pteridophyte plastome sequences, only three were from *Equisetum* species: one from *E. hyemale* [7] and two from US and Korean *E. arvense* [8,9], which were placed in subgenera *Equisetum* and *Hippochaete*, respectively e.g., [3,4,5,6]. These reports revealed plastome variation among *Equisetum* species. The two *E. arvense* genomes differed by 417 bp and the *E. hyemale* genome was about 1.5 kbp smaller than that of *E. arvense*. In addition, *rpl16* of *E. arvense* had an intron that is not present in *E. hyemale* [7,8,9]. These observations indicate that additional chloroplast genomes would be useful in evaluating evolutionary trends in this long-enduring genus.

Previous *Equisetum* plastome information was obtained using PCR amplification or from organelle-enriched DNA. The advancement of sequencing technologies and computational techniques allowed us to obtain complete organelle genome sequences from the shotgun metagenomic data we have archived for *E. xylochaetum*, presenting an additional technical option for obtaining *Equisetum* plastid genomes. Therefore, in this study, we assembled the complete plastid genome of *E. xylochaetum*, a giant species endemic to the Atacama Desert of Chile, South America, and used the information obtained to explore the evolution of *Equisetum* plastid genomes and the phylogenetic relationship of *Equisetum* species, as well as to determine whether the phylogeny estimated by using the popular plastid conserved regions was congruent with the haplotype mapping results of the corresponding sequences. Results showed that we successfully constructed the de novo plastid genome of *E. xylochaetum* using the shotgun metagenomic data. Phylogenetic estimations and comparison of *Equisetum* plastid genomes showed that *E. xylochaetum* was in the subgenus *Hippochaete* and that the *Equisetum* plastid genomes from subgenera *Hippochaete* and *Equisetum* were conserved in terms of genome structure, gene content, and gene order. Furthermore, results from TCS haplotype mapping showed that some of the taxa had a higher level of nucleotide diversity and some of the taxa shared common nucleotide haplotypes. Therefore, more conserved nucleotide regions and complete plastid genomes are needed for a better understanding of the evolutionary relationships of *Equisetum*. 

## 2. Results

**Figure 1:**
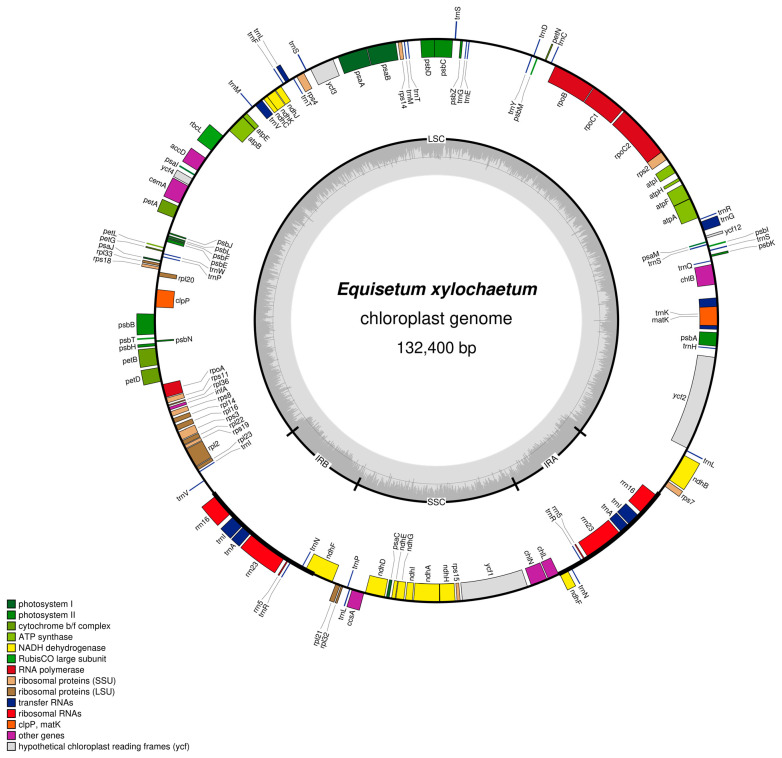
Circular map of the *Equisetum xylochaetum* plastid genome, NCBI accession MW282958, drawn by OGDRAW version 1.3.1 [10]. Genes positioned on the outside of the map are transcribed counterclockwise and those inside the map are transcribed clockwise. The thick lines indicate the extent of the inverted repeat regions.

The chloroplast genome of *E. xylochaetum* displayed a quadripartite structure. The single-copy regions were 93,902 bp and 9726 bp, with two reverse repeated regions (IRa and IRb) of 14,386 bp in length. The GC contents of the LSC, SSC, and IR regions individually, and of the cp genome as a whole, were 31.5%, 30.9%, 48.4%, and 33.9%, respectively. The *E. xylochaetum* plastome encoded a total of 119 unique genes, of which nine were duplicated in the IR regions. Seventy-eight were protein-coding genes, 38 were tRNA genes, and eight were rRNA genes. Fourteen genes contain introns (*atpF*, *clpP*, *ndhA*, *ndhB*, *petB*, petD, *rpl2*, *rpoC1*, *rps12*, *ycf3*, *trnK*(uuu), *trnL*(uaa), *trnV*(uac), and *trnI*(gau)) as shown in Figure 1. 

A comparison of *Equisetum* plastid genomes showed a collinear relationship, forming only one syntenic block in whole genome alignment. The genomes had similar genome size, % GC, gene content, gene length, and had identical gene order (Table 1 and Table 2). The protein-coding regions of *E. xylochaetum* plastid genes were subjected to purifying selection when compared against the corresponding protein-coding genes of *E. hyemale* and *E. arvense*.

**Figure 2:**
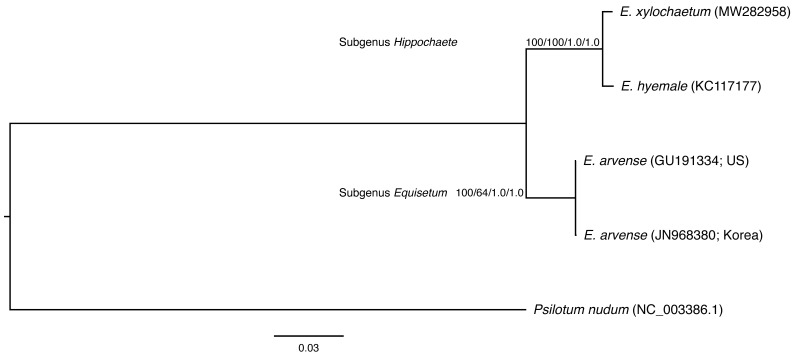
Maximum-likelihood tree inferred from all *Equisetum* plastome protein-coding regions using a GTR+I+F model. The scale bar represents the estimated number of nucleotide substitutions per site. The bootstrap and posterior probability values are reported at the respective nodes. The values include the ML bootstrap values of nucleotide and protein data and the BI posterior probability of the nucleotide and protein data, respectively.

Protein-coding regions of *Equisetum* species were similar in size, ranging between having the same length in *atpB*, *E*, *F*, *H*, *I*, *clpP*, *infA*, *ndhB*, *C*, *D*, *E*, *H*, *I*, *petB*, *D*, *G*, *L*, *N*, *psaA*, *B*, *C*, *I*, *J*, *M*, *psbA*, *B*, *C*, *D*, *E*, *F*, *H*, *I*, *J*, *K*, *L*, *M*, *N*, *Z*, *rbcL*, *rpl14*, *16*, *22*, *23*, *32*, *33*, *36*, *rps2*, *3*, *4*, *7*, *8*, *11*, *12*, *14*, *15*, *18*, and *19* to having a 195 bp or 64 amino acids difference in *accD*. These genes have a similar number and position of introns except for the presence of 753 bp of intron in *rpl16* in *E. arvense*. The percentage of the identical nucleotide of the aligned sites ranged from 88.7 percent in *matK* to 99.2 percent in *psbJ*, while the percentage of the identical derived amino acid of the aligned sites ranged from 73.9 percent in *atpF* to 100 percent in *atpH*, *ndhE*, *petB*, *D*, *G*, *N*, *psaJ*, *psbA*, *E*, *F*, *I*, *J*, *L*, *Z*, *rpl36*, and *rps2* (Table 1). Phylogenetic estimation of *Equisetum* using plastome protein-coding sequences suggested that the known complete plastid genomes of *Equisetum* species formed a monophyletic clade of the two subgenera, *Hippochaete* and *Equisetum*. The newly assembled *E. xylochaetum* plastome indicates placement within *Hippochaete* with *E. hyemale* (Figure 2).

**Figure 3:**
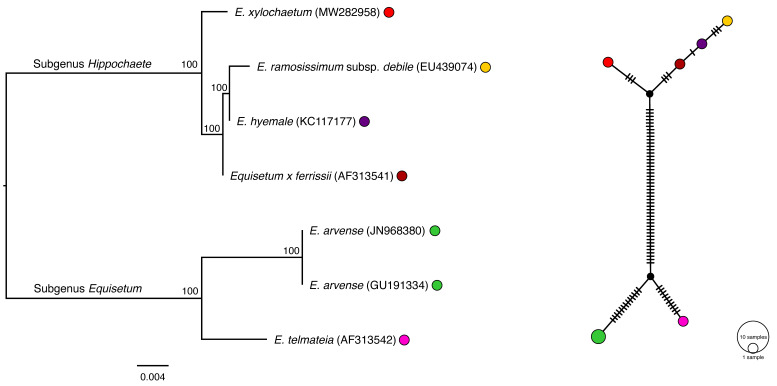
Phylogenetic estimation and TCS network of *Equisetum atpB* sequences. The scale bar of the tree represents the estimated number of nucleotide substitutions per site. The maximum-likelihood bootstrap values are reported at the respective nodes. The colours of taxa present in the tree correspond with the colours in the TCS haplotype map. The size of the circle represents the number of the taxa that share the same haplotype.

**Figure 4:**
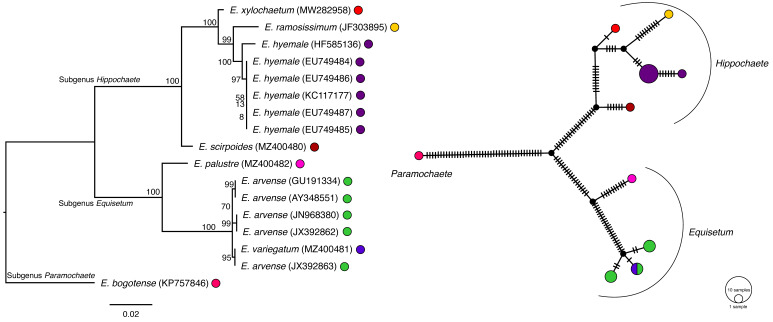
Phylogenetic estimation and TCS network of *Equisetum matK* sequences. The scale bar of the tree represents the estimated number of nucleotide substitutions per site. The maximum-likelihood bootstrap values are reported at the respective nodes. The colours of taxa present in the tree correspond with the colours in the TCS haplotype map. The size of the circle represents the number of the taxa that share the same haplotype.

**Figure 5:**
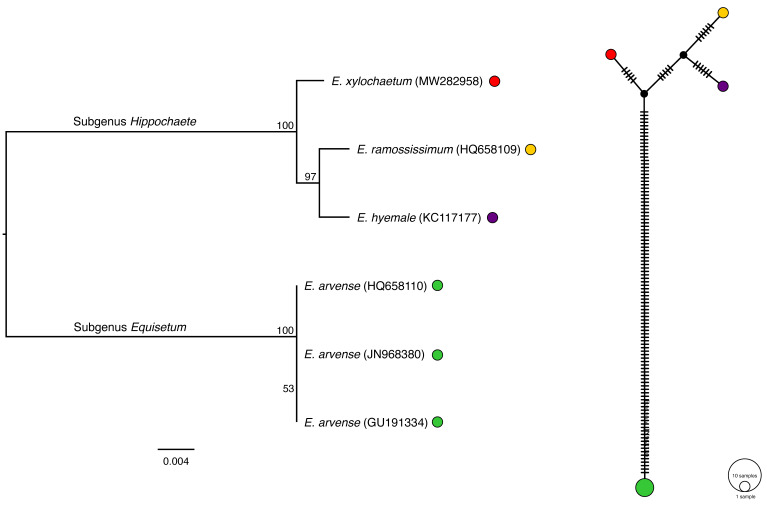
Phylogenetic estimation and TCS network of *Equisetum rpoB* sequences. The scale bar of the tree represents the estimated number of nucleotide substitutions per site. The maximum-likelihood bootstrap values are reported at the respective nodes. The colours of taxa present in the tree correspond with the colours in the TCS haplotype map. The size of the circle represents the number of the taxa that share the same haplotype.

**Figure 6:**
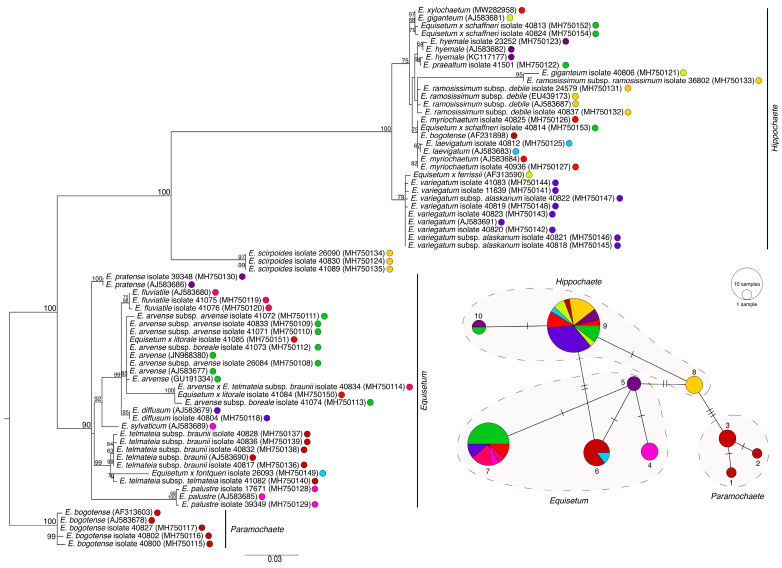
Phylogenetic estimation and TCS network of *Equisetum rps4* sequences. The scale bar of the tree represents the estimated number of nucleotide substitutions per site. The maximum-likelihood bootstrap values are reported at the respective nodes. The colours of taxa present in the tree correspond with the colours in the TCS haplotype map. The size of the circle represents the number of the taxa that share the same haplotype.

**Figure 7:**
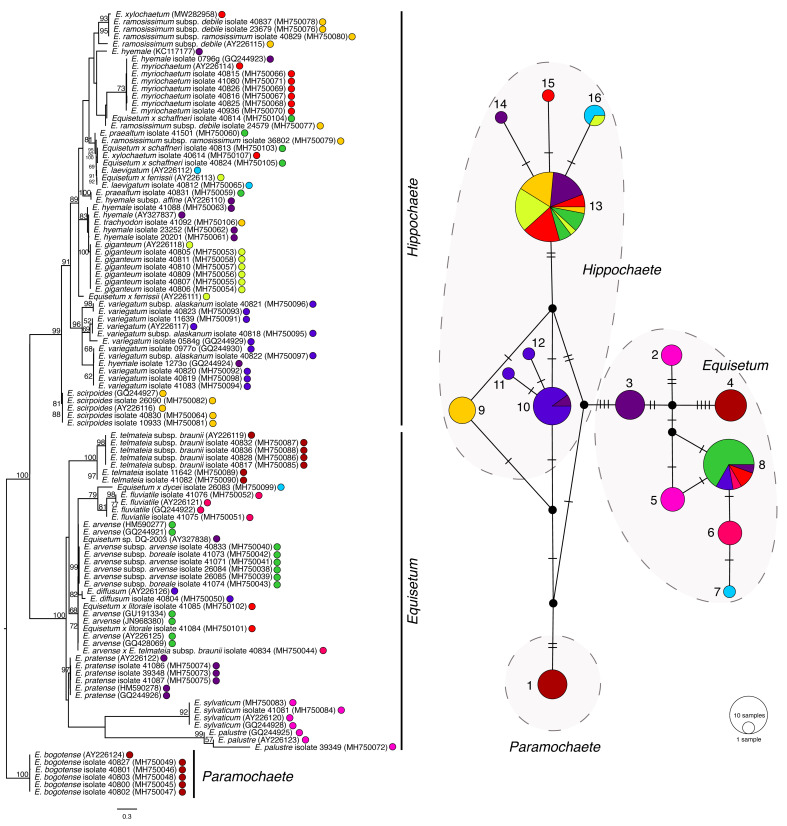
Phylogenetic estimation and TCS network of *Equisetum trnL-trnF*. The scale bar of the tree represents the estimated number of nucleotide substitutions per site. The maximum-likelihood bootstrap values are reported at the respective nodes. The colours of taxa present in the tree correspond with the colours in the TCS haplotype map. The size of the circle represents the number of the taxa that share the same haplotype.

Single-gene ML phylogenetic analysis of *atpB*, *matK*, *rpoB*, *rps4*, and *trnL-F* resolved the known subgenera of *Equisetum*, including *Paramochaete*, *Hippochaete*, and *Equisetum* (Figure 3, Figure 4, Figure 5, Figure 6 and Figure 7). The majority of the *Equisetum* species were resolved with ML bootstrap values of at least 50. However, the monophyly of some *Equisetum* species could not be resolved. The monophyly of *E. arvense* and *E. variegatum* was not resolved in the *matK* tree, the monophyly of *E. bogotense*, *E. laevigatum*, *E. myriochaetum*, *E. hyemale*, and *E. giganteum* was not resolved in the *rps4* tree, and the monophyly of *E. hyemale*, *E. praealtum*, *E. ramosissimum*, *E. trachyodon*, and *E. xylochaetum* was not resolved in the *trnL-F* tree. All hybrid taxa were phylogenetically placed within the clade consisting of the majority of their maternal parent, if the monophyly of the taxa was absent. In the case of the *rps4* tree, these hybrids included *Equisetum x fontqueri* isolate 26093 located within the clade of *E. telmateia*, *Equisetum x litorale* isolates 41084 and 41085 with *E. arvense*, *Equisetum x schaffneri* isolates 40813 and 40824 with *E. giganteum*, and *Equisetum x schaffneri* isolate 40814 with *E. myriochaetum*. For *trnL-F*, the hybrid taxa *Equisetum x ferrissii* (AY226113) located in the clade with *E. laevigatum*, *Equisetum x litorale* isolates 41084 and 41085 with *E. arvense*, and *Equisetum x schaffneri* isolate 40814 with *E. myriochaetum*.

TCS haplotype network analyses using *atpB*, *matK*, and *rpoB* resolved distinct clades representing each of the *Equisetum* subgenera. At the species level, haplotype networks constructed using *atpB* and *rpoB* showed one haplotype for each *Equisetum* species. In contrast, maps of *matK*, *rps4*, and *trnL-F* resolved more than one haplotype for some species and resolved some haplotypes that consisted of more than one species. For *matK*, there was more than one haplotype for *E. arvense* and *E. hyemale* and there was one haplotype that consisted of sequences from *E. arvense* and *E. variegatum* (Figure 4).

Haplotype maps of *rps4* and *trnL-F* seemed to be more complex compared to those of *atpB*, *matK*, and *rpoB*. In the map of *rps4* (Figure 6), we observed 10 haplotypes, of which, haplotypes 1–3 of *E. bogotense* appeared as a distinct group representing subgenus *Paramochaete*. A few haplotypes consisted of only one *Equisetum* species, which were haplotype 4 for *E. palustre*, haplotype 5 for *E. diffusum*, and haplotype 8 for *E. scirpoides*. The hybrid taxa were embedded within the same haplotypes as their maternal taxa. These included *Equisetum x fontqueri* isolate 26093 that was in haplotype 6 with *E. telmateia*, *Equisetum x litorale* isolates 41084 and 41085 in haplotype 7 with *E. arvense*, *Equisetum x schaffneri* isolates 40813 and 40824 in haplotype 9 with *E. giganteum*, and *Equisetum x schaffneri* isolate 40814 in haplotype 9 with *E. myriochaetum*. Some haplotypes consisted of many plant species, i.e., haplotype 7 and 9, where the majority of *Equisetum* and Hippochaete were placed together, respectively. Interestingly, a *rps4* sequence from *E. hyemale* grouped with other sequences of that species but also was present as a unique haplotype, as haplotype 10 with *E. praealtum* isolate 41501. 

The map of *trnL-F* (Figure 7) resolved two distinct groups of haplotypes representing subgenus *Paramochaete* (haplotype 1) and subgenus *Equisetum* (haplotypes 2–8). Many of the *Equisetum* species were present as unique haplotypes, including *E. bogotens*e (haplotype 1), *E. palustre* (haplotype 2), *E. pratense* (haplotype 3), *E. telmateia* (haplotype 4), *E. sylvaticum* (haplotype 5), *E. fluviatile* (haplotype 6), *Equisetum x dycei* (haplotype 7), and *E. scirpoides* (haplotype 9). 

Some *Equisetum* species were resolved as more than a single haplotype. *E. hyemale* isolate 20201 was resolved as a unique haplotype 14 while *E. hyemale* isolate 1273o was located in haplotype 10 with *E. variegatum*. For *E. variegatum*, in addition to its member in haplotype 10, *E. variegatum* isolates 40820 and 40823 were resolved as additional unique haplotypes 11 and 12. In addition, *E. myriochaetum* isolate 40826 was present as haplotype 15, while most members were located in haplotype 13. 

Most of the hybrid taxa in the *trnL-F* map were placed in the same haplotypes as their maternal taxa. *Equisetum x ferrissii* (AY226113) was located in haplotype 16 with its maternal taxon, *E. laevigatum*, *Equisetum x ferrissii* in haplotype 13 with the majority of *E. hyemale*, *Equisetum x litorale* isolates 41084 and 41085 in haplotype 8 with *E. hyemale*, *Equisetum x schaffneri* isolates 40813 and 40824 in haplotype 13 with *E. giganteum*, and *Equisetum x schaffneri* isolate 40814 in haplotype 13 with the majority of *E. myriochaetum*.

## 3. Discussion

In this study, we assembled the complete plastid genome of *E. xylochaetum* from shotgun metagenomes of *E. xylochaetum* sampled from two Atacama Desert locales exhibiting different degrees of disturbance. Results showed that the plastid genomes constructed from these two *E. xylochaetum* metagenome accessions were identical, suggesting that the *Equisetum* samples were from the same *Equisetum* population. Comparison of nucleotide, and their derived protein, sequences of this newly assembled *E. xylochaetum* plastid genome to those of *E. hyemale* and *E. arvense* showed that the *Equisetum* plastid genomes were highly conserved in terms of structure and function, even though the two subgenera (*Hippochaete* and *Equisetum*) might have diverged as early as 135 Mya during the early Cretaceous [4,6]. All the plastid protein-coding sequences were subjected to purifying selection, with genes of the same type having identical nucleotide percentages and having nucleotide identity ranging from 88.7–99.2 percent. The only major difference in gene structure was presence of the intron in *E. arvense rpl16*. To determine where and when the intron of *rp116* originated in the *Equisetum* lineage, more *Equisetum rpl16* sequences or complete plastid genomes are required.

In a broad sense, the phylogenetic positions of *Equisetum* species inferred by using all protein-coding sequences along with their derived proteins and the single-gene analysis present in this study were congruent with results from previous studies that used a single-gene approach [11] or a combination of multi-genes and morphological characters e.g., [4,5,6], where *Equisetum* formed monophyletic clades of each subgenus and placed *E. xylochaetum* in *Hippochaete*. Despite the presence of the high conservation level of *Equisetum* plastid genes, it was surprising to us that the single-gene phylogenetic approach was not sufficient to resolve relationships among *Equisetum* taxa, especially those closely related taxa placed in subgenus *Hippochaete*, e.g., *E. giganteum*, *E. variegatum*, and *E. hyemale*. Therefore, it is evident that more *Equisetum* plastid genomes, plus additional molecular information from other genetic compartments, are needed. 

The addition of haplotype mapping provided in this study enhanced the understanding of how plastid genes from each taxon are related. In general, the haplotype maps reflected the relationship resolved from phylogenetic estimation using the corresponding nucleotide regions. Even so, these new maps aid the visualisation of how these plastome nucleotide data were interrelated to each other at the level of isolate, species, and subgenus. The presence of only one shared distinct haplotype of an *Equisetum* species, though its samples were collected from different locales, suggested a high conservation level of the corresponding genes within its plastid genomes. On the other hand, the presence of more than one haplotype at the specific level suggested the presence of nucleotide diversity, indicating the need to further examine the populations of *E. arvense*, *E. bogotense*, *E. hyemale*, *E. variegatum*, and *E. myriochaetum*. In addition, the presence of a haplotype consisting of more than one *Equisetum* species, e.g., haplotypes 8 and 9 of the *rps4* map and haplotypes 8 and 13 of the *trnL-F* map, suggested that these conserved regions alone were not sufficient for studying the relationship and diversity of *Equisetum* taxa. These findings emphasize the need for more *Equisetum* plastid genomes.

The presence of distinct haplotype(s) in the early-diverging species *E. bogotense* in *rps4* and *trnL-F* suggested that these plastid sequences might not represent the ancestral characters of *Equisetum*. Instead, these *E. bogotense* samples may only represent the survival representatives of the extinct members that also evolved during the course of time. In contrast, according to the *rps4* and *trnL-F* maps, the taxa that frequently occurred at the junction region between each subgenus were *E. scirpoides* and *E. pratense*, suggesting that these taxa might be particularly helpful for understanding how the *Equisetum* subgenera diverged. 

## 4. Materials and Methods

Nucleotide data for *Equisetum xylochaetum* Mett. were obtained from GenBank BioProject PRJNA555713 [12], generated by metagenomic shotgun sequencing of the microbiome of giant *Equisetum xylochaetum* sampled from two streambed locales in the Atacama Desert of northern Chile that differed in the degree of human disturbance. The two raw data sets, separately archived in accessions SRX6486516 and SRX6486517, each represented pooled replicate DNA extractions from both above-ground green and below-ground non-green tissues. To obtain the complete chloroplast genome of *E. xylochaetum*, metagenomic sequences were trimmed using Trimmomatic v. 0.39 [13] using the parameter sliding window:4:30. Next, the trimmed sequences from the two raw data sets were independently assembled using MEGAHIT ver. 1.2.9 [14] with the parameter “bubble-level equal to 0” in order to prevent the merging of sequences that were highly similar, e.g., sequences from closely related species or sequences that display single nucleotide polymorphisms. Each assembly yielded a contig of the complete plastid genome of *E. xylochaetum*, and these two contigs were identical in sequence. To validate the assembly, we calculated the coverage of the plastid genomes using the methods described in Satjarak and Graham [15]. One of the two contigs, which had the mean coverage of 706 fold, was then selected for annotation of protein-coding genes using proteins inferred from *E. arvense* [8,9] and *E. hyemale* [7] as references. The tRNAs and rRNA genes were annotated using tRNAscan-SE On-line [16] and the RNAmmer 1.2 Server [17], respectively. The complete plastid genome of *E. xylochaetum* was deposited in GenBank under accession number MW282958. A representative plant specimen has been deposited at the University of Concepción herbarium under accession number CONC-CH 6005. 

We compared the plastid genome of *E. xylochaetum* obtained from this study to other complete *Equisetum* plastid genomes, including *E. hyemale* (KC117177), *E. arvense* from the US (GU191334), and *E. arvense* from Korea (JN968380). We examined general characteristics of the genomes, including the genome size, %GC, the gene content, gene length, gene order, and polymorphism of nucleotides within coding regions and their derived proteins. To consider nucleotide polymorphisms, we aligned the protein-coding sequences (Table 1) using Geneious translation alignment: global alignment with free end gap, standard genetic code, and Identity (1.0/0.0) cost matrix (Geneious ver. 9.1.3; https://www.geneious.com; accessed in 31 January 2022). 

The mode of evolution of protein-coding regions was performed using the method described in Mekvipad and Satjarak [18]. For the polymorphism of protein sequences, we aligned the derived protein sequences using MAFFT alignment: auto algorithm and Blosum62 scoring matrix [19]. To investigate the relationship of *E. xylochaetum* and other *Equisetum* complete chloroplast genomes, *Psilotum nudum* (NC_003386.1) was used as an outgroup. The protein-coding sequences and protein sequences (Table 1) were similarly aligned, trimmed using Trimal ver. 1.2 [20], and concatenated. The nucleotide data matrix was 60,987 bp and the protein data matrix consisted of 19,435 amino acids. Phylogenetic relationships were estimated using maximum likelihood and Bayesian frameworks as described in Satjarak and Graham [15]. 

To investigate whether the *Equisetum* relationship resolved from frequently-used nucleotide sequences reported in previous studies exhibited grades or evolutionary intermediates, we performed haplotype network analysis of selected, frequently-used *Equisetum* conserved regions. These included *atpB*, *matK*, *rpoB*, *rps4*, and *trnL-F* (Table 3). To prepare the data matrices, the conserved nucleotide regions were extracted from the complete plastid genomes and from DNA sequences from other published studies (Table 3). Next, the data were aligned, and the phylogenetic trees were estimated using the methods described above. The haplotype network analysis was calculated using (Templeton, Crandall, and Sing; TCS) [21] and visualized in PopArt v1.7 [22]. 

## 5. Conclusions

In summary, our study demonstrated that metagenomic data can be a useful way to obtain plastid genomes. The comparison of the de novo plastid genome of *E. xylochaetum* with other reported *Equisetum* plastomes showed a high degree of conservation in terms of structure, gene content, gene order, and nucleotide polymorphisms. Even so, this new plastid genome provided additional information about the evolution and diversity of *Equisetum*, e.g., the presence of an intron in *rpl16*. Haplotype analyses of the selected conserved nucleotides showed that some *Equisetum* species were distantly related to other taxa, inferred from the presence of distinct haplotypes. Many of the taxa appeared as shared haplotypes, suggesting that the molecular data we currently have might not be sufficient for a full understanding of the evolutionary relationship of *Equisetum* and that more *Equisetum* plastid genomes are needed.

## Figures and Tables

**Table 1:** Comparison of general characters of *Equisetum* plastid genomes.

	*E. xylochaetum*	*E. hyemale*	*E. arvense* (US)	*E. arvense* (Korea)
**Accession**	MW282958	KC117177	GU191334	JN968380
**genome size**	132,400	131,760	133,309	132,726
**LSC**	93,902	92,580	93,542	92,961
**SSC**	9,726	18,994	19,469	19,477
**IRs**	14,386	10,093	10,149	10,144
**%GC**	33.9	33.7	33.4	33.4

**Table 2:** Protein-coding gene content and introns of *Equisetum* plastid genomes. Comparison showed percent identity and size of the gene and its derived proteins.

		DNA					Protein				
No.	Gene (# of Intron)	Identical Site (%)	Mean (bp)	SD (bp)	min (bp)	max (bp)	Identical Site (%)	Mean (aa)	SD (aa)	min (aa)	max (aa)
1.	*accD*	92.8	1021.5	81	948	1143	89.9	340	26.5	316	380
2.	*atpA*	95.5	1539	20.8	1527	1575	98	512	6.9	508	524
3.	*atpB*	94.4	1470	0	1470	1470	96.3	489	0	489	489
4.	*atpE*	93.4	396	0	396	396	93.1	131	0	131	131
5.	*atpF* (1)	97.5	555	0	555	555	73.9	184	0	184	184
6.	*atpH*	96.3	246	0	246	246	100	81	0	81	81
7.	*atpI*	95.4	747	0	747	747	99.1	248	0	284	248
8.	*ccsA*	93.3	943.5	1.5	942	945	91.1	313.5	0.5	313	314
9.	*cemA*	89.5	1438.5	21.7	1425	1476	82.7	478.5	7.2	474	491
10.	*chlB*	94.3	1549.5	4.5	1545	1554	93.2	515.5	1.5	514	517
11.	*chlL*	91.6	879	6	873	885	93.9	292	2	290	294
12.	*chlN*	92.8	1300.5	10.5	1290	1311	90.4	432.5	3.5	429	436
13.	*clpP* (1)	95.1	615	0	615	615	98.5	204	0	204	204
14.	*infA*	94.7	243	0	243	243	96.3	80	0	80	80
15.	*matK*	88.7	1470	3	1467	1473	81.4	489	1	488	490
16.	*ndhA* (1)	92.7	1101.8	1.3	1101	1104	91.8	366.3	0.4	366	367
17.	*ndhB* (1)	94.8	1473	0	1473	1473	94.3	490	0	490	490
18.	*ndhC*	95.9	363	0	363	363	98.3	120	0	120	120
19.	*ndhD*	95.1	1497	0	1497	1497	95.2	498	0	498	498
20.	*ndhE*	98.3	303	0	303	303	100	100	0	100	100
21.	*ndhF*	92.8	2221.5	1.5	2220	2223	92.2	739.5	0.5	739	740
22.	*ndhG*	91.4	606	17.2	585	633	85.7	201	5.7	194	210
23.	*ndhH*	95.3	1182	0	1182	1182	97.2	393	0	393	393
24.	*ndhI*	97.3	549	0	549	549	98.4	182	0	182	182
25.	*ndhJ*	93.9	520.5	4.5	516	525	94.8	172.5	1.5	171	174
26.	*ndhK*	90.6	747.8	9.1	732	753	86.8	248.3	3	243	250
27.	*petA*	92.4	955.5	7.5	948	963	93.8	317.5	2.5	315	320
28.	*petB* (1)	96	648	0	648	648	100	215	0	215	215
29.	*petD* (1)	96.9	483	0	483	483	100	160	0	160	160
30.	*petG*	97.4	114	0	114	114	100	37	0	37	37
31.	*petL*	93.8	96	0	96	96	93.5	31	0	31	31
32.	*petN*	99	96	0	96	96	100	31	0	31	31
33.	*psaA*	96.2	2253	0	2253	2253	99.6	750	0	750	750
34.	*psaB*	95.7	2205	0	2205	2205	99.2	734	0	734	734
35.	*psaC*	95.1	246	0	246	246	98.8	81	0	81	81
36.	*psaI*	91.9	111	0	111	111	94.4	36	0	36	36
37.	*psaJ*	97.7	129	0	129	129	100	42	0	42	42
38.	*psaM*	96	99	0	99	99	96.9	32	0	32	32
39.	*psbA*	98.1	1062	0	1062	1062	100	353	0	353	353
40.	*psbB*	96.1	1527	0	1527	1527	99	508	0	508	508
41.	*psbC*	95	1422	0	1422	1422	99.4	473	0	473	473
42.	*psbD*	95.6	1062	0	1062	1062	87.3	353	0	353	353
43.	*psbE*	97.2	246	0	246	246	100	81	0	81	81
44.	*psbF*	98.3	120	0	120	120	100	39	0	39	39
45.	*psbH*	94.7	225	0	225	225	89.2	74	0	74	74
46.	*psbI*	97.3	111	0	111	111	100	36	0	36	36
47.	*psbJ*	99.2	123	0	123	123	100	40	0	40	40
48.	*psbK*	97	168	0	168	168	96.4	55	0	55	55
49.	*psbL*	98.3	117	0	117	117	100	38	0	38	38
50.	*psbM*	98.2	111	0	111	111	94.4	36	0	36	36
51.	*psbN*	95.5	132	0	132	132	93	43	0	43	43
52.	*psbT*	97.4	112.5	1.5	111	114	97.3	36.5	0.5	36	37
53.	*psbZ*	94.2	189	0	189	189	100	62	0	62	62
54.	*rbcL*	96.1	1428	0	1428	1428	99.2	475	0	475	475
55.	*rpl14*	97.6	369	0	369	369	99.2	122	0	122	122
56.	*rpl16* (1 in *E. arvense*)	93.1	423	0	423	423	95	140	0	140	140
57.	*rpl2* (1)	94.3	834.8	1.3	834	837	95.3	277.3	0.4	277	278
58.	*rpl20*	89.7	347.3	1.3	345	348	85.2	114.8	0.4	114	115
59.	*rpl21*	91.3	364.5	1.5	363	366	86	120.5	0.5	120	121
60.	*rpl22*	94.1	372	0	372	372	96.6	123	0	123	123
61.	*rpl23*	94.9	273	0	273	273	93.3	90	0	90	90
62.	*rpl32*	95.3	171	0	171	171	98.2	56	0	56	56
63.	*rpl33*	95.5	201	0	201	201	90.9	66	0	66	66
64.	*rpl36*	93.9	114	0	114	114	100	37	0	37	37
65.	*rpoA*	93.5	1.18.5	4.5	1014	1023	93.5	338.5	1.5	337	340
66.	*rpoB*	93.9	3235.5	33.8	3216	3294	92.9	1.00.5	11.3	1071	1097
67.	*rpoC1* (1)	93.3	2060.3	3.9	2058	2067	91.3	685.8	1.3	685	688
68.	*rpoC2*	92.3	4143	21	4122	4164	87.2	1380	7	1373	1387
69.	*rps11*	94.7	396	0	396	396	95.4	131	0	131	131
70.	*rps12*	98.1	372	0	372	372	100	123	0	123	123
71.	*rps14*	92.2	306	0	306	306	93.1	101	0	101	101
72.	*rps15*	95.2	270	0	270	270	92.1	89	0	89	89
73.	*rps18*	96.5	228	0	228	228	98.7	75	0	75	75
74.	*rps19*	95.7	279	0	279	279	98.9	92	0	92	92
75.	*rps2*	95.2	708	0	708	708	97	235	0	235	235
76.	*rps3*	95.4	657	0	657	657	96.3	218	0	218	218
77.	*rps4*	94.1	624	0	624	624	92.3	207	0	207	207
78.	*rps7*	94.9	468	0	468	468	94.8	155	0	155	155
79.	*rps8*	95.7	399	0	399	399	95.5	132	0	132	132

**Table 3:** Nucleotide sequences used in the single gene phylogenetic analysis and TCS haplotype mapping.

No.	Name	GenBank Accession	Locality	References
	***atpB***			
1.	*E. arvense*	GU191334	USA	[8]
2.	*E. arvense*	JN968380	Korea	[9]
3.	*E. hyemale*	KC117177	unknown	[7]
4.	*E. ramosissimum* subsp. *debile*	EU439074	unknown	[23]
5.	*E. telmateia*	AF313542	unknown	[24]
6.	*E. xylochaetum*	MW282958	Chile	This study
7.	*Equisetum x ferrissii*	AF313541	unknown	[24]
	***matK***			
1.	*E. arvense*	JX392862	China	[25]
2.	*E. arvense*	JX392863	Europe	[25]
3.	*E. arvense*	AY348551	unknown	[26]
4.	*E. arvense*	GU191334	USA	[8]
5.	*E. arvense*	JN968380	Korea	[9]
6.	*E. bogotense*	KP757846	unknown	[27]
7.	*E. hyemale*	EU749486	unknown	[28]
8.	*E. hyemale*	EU749485	unknown	[28]
9.	*E. hyemale*	EU749484	unknown	[28]
10.	*E. hyemale*	EU749487	unknown	[28]
11.	*E. hyemale*	HF585136	unknown	[29]
12.	*E. hyemale*	KC117177	unknown	[7]
13.	*E. palustre*	MZ400482	Sweden	[30]
14.	*E. ramosissimum*	JF303895	unknown	[31]
15.	*E. scirpoides*	MZ400480	Sweden	[30]
16.	*E. variegatum*	MZ400481	Sweden	[30]
17.	*E. xylochaetum*	MW282958	Chile	This study
	***rpoB***			
1.	*E. arvense*	HQ658110	China	[32]
2.	*E. arvense*	GU191334	USA	[8]
3.	*E. arvense*	JN968380	Korea	[9]
4.	*E. hyemale*	KC117177	Unknown	[7]
5.	*E. ramossissimum*	HQ658109	China	[32]
6.	*E. xylochaetum*	MW282958	Chile	This study
	***rps4***			
1.	*E. arvense* subsp. *arvense* isolate 41072	MH750111	Finland	[5]
2.	*E. arvense*	AJ583677	unknown	[3]
3.	*E. arvense*	JN968380	Korea	[9]
4.	*E. arvense*	GU191334	USA	[8]
5.	*E. arvense* subsp. *arvense* isolate 26084	MH750108	India (Himachal Pradesh)	[5]
6.	*E. arvense* subsp. *arvense* isolate 40833	MH750109	USA (California)	[5]
7.	*E. arvense* subsp. *arvense* isolate 41071	MH750110	Finland	[5]
8.	*E. arvense* subsp. *boreale* isolate 41073	MH750112	Finland/Norway (border)	[5]
9.	*E. arvense* subsp. *boreale* isolate 41074	MH750113	Finland/Norway (border)	[5]
10.	*E. arvense* x *E. telmateia* subsp. *braunii* isolate 40834	MH750114	USA (California)	[5]
11.	*E. bogotense*	AF231898	unknown	[33]
12.	*E. bogotense*	AF313603	unknown	[24]
13.	*E. bogotense*	AJ583678	unknown	[3]
14.	*E. bogotense* isolate 40800	MH750115	Argentina	[5]
15.	*E. bogotense* isolate 40802	MH750116	Ecuador	[5]
16.	*E. bogotense* isolate 40827	MH750117	Colombia	[5]
17.	*E. diffusum*	AJ583679	unknown	[3]
18.	*E. diffusum* isolate 40804	MH750118	India	[5]
19.	*E. fluviatile*	AJ583680	unknown	[3]
20.	*E. fluviatile* isolate 41075	MH750119	Finland	[5]
21.	*E. fluviatile* isolate 41076	MH750120	Finland	[5]
22.	*E. giganteum*	AJ583681	unknown	[3]
23.	*E. giganteum* isolate 40806	MH750121	Chile	[5]
24.	*E. hyemale*	AJ583682	unknown	[3]
25.	*E. hyemale*	KC117177	unknown	[7]
26.	*E. hyemale* isolate 23252	MH750123	Norway	[5]
27.	*E. laevigatum*	AJ583683	unknown	[3]
28.	*E. laevigatum* isolate 40812	MH750125	USA (California)	[5]
29.	*E. myriochaetum*	AJ583684	unknown	[3]
30.	*E. myriochaetum* isolate 40825	MH750126	Mexico	[5]
31.	*E. myriochaetum* isolate 40936	MH750127	El Salvador	[5]
32.	*E. palustre*	AJ583685	unknown	[3]
33.	*E. palustre* isolate 17671	MH750128	UK (England, Norfolk)	[5]
34.	*E. palustre* isolate 39349	MH750129	UK (England, Surrey)	[5]
35.	*E. praealtum* isolate 41501	MH750122	USA (Ohio)	[5]
36.	*E. pratense*	AJ583686	unknown	[3]
37.	*E. pratense* isolate 39348	MH750130	Finland	[5]
38.	*E. ramosissimum* subsp. *debile*	AJ583687	unknown	[3]
39.	*E. ramosissimum* subsp. *debile*	EU439173	unknown	[23]
40.	*E. ramosissimum* subsp. *debile* isolate 24579	MH750131	Sri Lanka	[5]
41.	*E. ramosissimum* subsp. *debile* isolate 40837	MH750132	New Caledonia	[5]
42.	*E. ramosissimum* subsp. *ramosissimum* isolate 36802	MH750133	Spain (Andalucia)	[5]
43.	*E. scirpoides*	AJ583688	unknown	[3]
44.	*E. scirpoides* isolate 26090	MH750134	Greenland	[5]
45.	*E. scirpoides* isolate 40830	MH750124	Russia (Kamtschatka)	[5]
46.	*E. sylvaticum*	AJ583689	unknown	[3]
47.	*E. telmateia* subsp. *braunii*	AJ583690	unknown	[3]
48.	*E. telmateia* subsp. *braunii* isolate 40817	MH750136	USA (California)	[5]
49.	*E. telmateia* subsp. *braunii* isolate 40828	MH750137	Canada (British Columbia)	[5]
50.	*E. telmateia* subsp. *braunii* isolate 40832	MH750138	USA (California)	[5]
51.	*E. telmateia* subsp. *braunii* isolate 40836	MH750139	USA (California)	[5]
52.	*E. telmateia* subsp. *telmateia* isolate 41082	MH750140	Ireland	[5]
53.	*E. variegatum*	AJ583691	unknown	[3]
54.	*E. variegatum* isolate 11639	MH750141	UK (Wales)	[5]
55.	*E. variegatum* isolate 40819	MH750148	Ireland	[5]
56.	*E. variegatum* isolate 40820	MH750142	France (Pyrenees)	[5]
57.	*E. variegatum* isolate 40823	MH750143	USA (Keweenaw, Michigan)	[5]
58.	*E. variegatum* isolate 41083	MH750144	Ireland	[5]
59.	*E. variegatum* subsp. *alaskanum* isolate 40818	MH750145	USA (Alaska)	[5]
60.	*E. variegatum* subsp. *alaskanum* isolate 40821	MH750146	Canada (British Columbia)	[5]
61.	*E. variegatum* subsp. *alaskanum* isolate 40822	MH750147	Canada (Banff)	[5]
62.	*E. xylochaetum*	MW282958	Chile	This study
63.	*Equisetum scirpoides* isolate 41089	MH750135	Finland	[5]
64.	*Equisetum x ferrissii*	AF313590	unknown	[24]
65.	*Equisetum x fontqueri isolate* 26093 *(E. telmateia x *E. palustre*)*	MH750149	UK (Scotland)	[5]
66.	*Equisetum x litorale* isolate 41084 *(E. arvense x *E. fluviatile*)*	MH750150	Ireland	[5]
67.	*Equisetum x litorale* isolate 41085 *(E. arvense x *E. fluviatile*)*	MH750151	Ireland	[5]
68.	*Equisetum x schaffneri* isolate 40813 *(E. giganteum x *E. myriochaetum*)*	MH750152	Mexico	[5]
69.	*Equisetum x schaffneri* isolate 40814 *(E. myriochaetum x *E. giganteum*)*	MH750153	Peru (cult RBG Edinburgh)	[5]
70.	*Equisetum x schaffneri* isolate 40824 *(E. giganteum x *E. myriochaetum*)*	MH750154	Mexico	[5]
	***trnL-trnF***			
1.	*E. arvense*	JN968380	Korea	[9]
2.	*E. arvense*	GU191334	USA	[8]
3.	*E. arvense*	AY226125	Franc	[34]
4.	*E. arvense*	GQ428069	unknown	[35]
5.	*E. arvense*	HM590277	Estonia	[36]
6.	*E. arvense*	GQ244921	unknown	[37]
7.	*E. arvense subsp boreale* isolate 41074	MH750043	Finland/Norway	[5]
8.	*E. arvense subsp. arvense* isolate 26084	MH750038	India	[5]
9.	*E. arvense subsp. arvense* isolate 26085	MH750039	UK	[5]
10.	*E. arvense subsp. arvense* isolate 40833	MH750040	USA	[5]
11.	*E. arvense subsp. arvense* isolate 41071	MH750041	Finland	[5]
12.	*E. arvense subsp. boreale* isolate 41073	MH750042	Finland/Norway	[5]
13.	*E. arvense x *E. telmateia** subsp. *braunii* isolate 40834	MH750044	USA	[5]
14.	*E. bogotense*	AY226124	Colombia	[34]
15.	*E. bogotense* isolate 40800	MH750045	Argentina	[5]
16.	*E. bogotense* isolate 40801	MH750046	Chile	[5]
17.	*E. bogotense* isolate 40802	MH750047	Ecuador	[5]
18.	*E. bogotense* isolate 40803	MH750048	Chile	[5]
19.	*E. bogotense* isolate 40827	MH750049	Colombia	[5]
20.	*E. diffusum*	AY226126	India	[34]
21.	*E. diffusum* isolate 40804	MH750050	India	[5]
22.	*E. fluviatile*	AY226121	Canada	[34]
23.	*E. fluviatile*	GQ244922	unknown	[37]
24.	*E. fluviatile* isolate 41075	MH750051	Finland	[5]
25.	*E. fluviatile* isolate 41076	MH750052	Finland	[5]
26.	*E. giganteum*	AY226118	Ecuador	[34]
27.	*E. giganteum* isolate 40805	MH750053	Jamaica	[5]
28.	*E. giganteum* isolate 40806	MH750054	Chile	[5]
29.	*E. giganteum* isolate 40807	MH750055	Peru	[5]
30.	*E. giganteum* isolate 40810	MH750057	Argentina	[5]
31.	*E. giganteum* isolate 40811	MH750058	Argentina	[5]
32.	*E. hyemale*	KC117177	unknown	[7]
33.	*E. hyemale*	AY327837	unknown	[34]
34.	*E. hyemale* isolate 0796g	GQ244923	unknown	[37]
35.	*E. hyemale* isolate 1273o	GQ244924	unknown	[37]
36.	*E. hyemale* isolate 20201	MH750061	France	[5]
37.	*E. hyemale* isolate 23252	MH750062	Norway	[5]
38.	*E. hyemale* isolate 41088	MH750063	Finland	[5]
39.	*E. hyemale* subsp. *affine*	AY226110	USA	[34]
40.	*E. iganteum* isolate 40809	MH750056	Argentina	[5]
41.	*E. laevigatum*	AY226112	USA	[34]
42.	*E. laevigatum* isolate 40812	MH750065	USA	[5]
43.	*E. myriochaetum*	AY226114	USA	[34]
44.	*E. myriochaetum* isolate 40815	MH750066	USA	[5]
45.	*E. myriochaetum* isolate 40816	MH750067	USA	[5]
46.	*E. myriochaetum* isolate 40825	MH750068	Mexico	[5]
47.	*E. myriochaetum* isolate 40826	MH750069	Ecuador	[5]
48.	*E. myriochaetum* isolate 40936	MH750070	El Savador	[5]
49.	*E. myriochaetum* isolate 41080	MH750071	Guatemala	[5]
50.	*E. palustre*	AY226123	Canada	[34]
51.	*E. palustre*	GQ244925	unknown	[37]
52.	*E. palustre* isolate 39349	MH750072	UK	[5]
53.	*E. praealtum* isolate 40831	MH750059	USA	[5]
54.	*E. praealtum* isolate 41501	MH750060	USA	[5]
55.	*E. pratense*	AY226122	Canada	[34]
56.	*E. pratense*	GQ244926	unknown	[37]
57.	*E. pratense*	HM590278	Estonia	[36]
58.	*E. pratense* isolate 39348	MH750073	Finland	[5]
59.	*E. pratense* isolate 41086	MH750074	Finland	[5]
60.	*E. pratense* isolate 41087	MH750075	Finland	[5]
61.	*E. ramosissimum* subsp. *debile*	AY226115	Taiwan	[34]
62.	*E. ramosissimum* subsp. *debile* isolate 23679	MH750076	Reunion	[5]
63.	*E. ramosissimum* subsp. *debile* isolate 24579	MH750077	Sri Lanka	[5]
64.	*E. ramosissimum* subsp. *debile* isolate 40837	MH750078	New Caledonia	[5]
65.	*E. ramosissimum* subsp. *ramosissimum* isolate 36802	MH750079	Spain	[5]
66.	*E. ramosissimum* subsp. *ramosissimum* isolate 40829	MH750080	Turkey	[5]
67.	*E. scirpoides*	AY226116	Canada	[34]
68.	*E. scirpoides*	GQ244927	unknown	[37]
69.	*E. scirpoides* isolate 26090	MH750082	Greenland	[5]
70.	*E. scirpoides* isolate 40830	MH750064	Russia	[5]
71.	*E. scirpoides* isolate10933	MH750081	UK	[5]
72.	*E. sylvaticum*	MH750083	UK	[5]
73.	*E. sylvaticum*	AY226120	France	[34]
74.	*E. sylvaticum*	GQ244928	unknown	[37]
75.	*E. sylvaticum* isolate 41081	MH750084	Finland	[5]
76.	*E. telmateia* isolate 11642	MH750089	China	[5]
77.	*E. telmateia* isolate 41082	MH750090	Ireland	[5]
78.	*E. telmateia* subsp. *braunii*	AY226119	USA	[34]
79.	*E. telmateia* subsp. *braunii* isolate 40817	MH750085	USA	[5]
80.	*E. telmateia* subsp. *braunii* isolate 40828	MH750086	Canada	[5]
81.	*E. telmateia* subsp. *braunii* isolate 40832	MH750087	USA	[5]
82.	*E. telmateia* subsp. *braunii* isolate 40836	MH750088	USA	[5]
83.	*E. trachyodon* isolate 41092	MH750106	Finland	[5]
84.	*E. variegatum*	AY226117	USA	[34]
85.	*E. variegatum* isolate 0584g	GQ244929	unknown	[37]
86.	*E. variegatum* isolate 0977o	GQ244930	unknown	[37]
87.	*E. variegatum* isolate 11639	MH750091	UK	[5]
88.	*E. variegatum* isolate 40819	MH750098	Ireland	[5]
89.	*E. variegatum* isolate 40820	MH750092	France	[5]
90.	*E. variegatum* isolate 40823	MH750093	USA	[5]
91.	*E. variegatum* isolate 41083	MH750094	Ireland	[5]
92.	*E. variegatum* subsp. *alaskanum* isolate 40818	MH750095	USA	[5]
93.	*E. variegatum* subsp. *alaskanum* isolate 40821	MH750096	Canada	[5]
94.	*E. variegatum* subsp. *alaskanum* isolate 40822	MH750097	Canada	[5]
95.	*E. xylochaetum*	MW282958	Chile	This study
96.	*E. xylochaetum* isolate 40614	MH750107	Chile	[5]
97.	*Equisetum sp.*	AY327838	unknown	[34]
98.	*Equisetum x dycei* isolate 26083	MH750099	UK	[5]
99.	*Equisetum x ferrissii* (*E. laevigatum x *E. hyemale**)	AY226113	USA	[34]
100.	*Equisetum x ferrissii* (*Equisetum hyemale x laevigatum*)	AY226111	Canada	[34]
101.	*Equisetum x litorale* isolate 41084	MH750101	Ireland	[5]
102.	*Equisetum x litorale* isolate 41085	MH750102	Ireland	[5]
103.	*Equisetum x schaffneri* isolate 40813	MH750103	Mexico	[5]
104.	*Equisetum x schaffneri* isolate 40814	MH750104	Peru	[5]
105.	*Equisetum x schaffneri* isolate 40824	MH750105	Mexico	[5]

## Data Availability

The complete plastid genome of *Equisetum xylochaetum* is publicly available in NCBI GenBank (https://www.ncbi.nlm.nih.gov accessed on 12 March 2022) accession number MW282958. Nucleotide data for analysis are available at GenBank BioProject PRJNA555713 accessions SRX6486516 and SRX6486517.
